# Different approaches to selection of surgical trainees in the European Union

**DOI:** 10.1186/s12909-021-02779-5

**Published:** 2021-06-30

**Authors:** Kristine Hagelsteen, Hanne Pedersen, Anders Bergenfelz, Chris Mathieu

**Affiliations:** 1 Practicum Clinical Skills Centre, Department of Clinical Sciences Lund, Lund University, Skane University Hospital, Lund, Sweden; 2 Department of Sociology, Faculty of Social Sciences, Lund University, Lund, Sweden

**Keywords:** Admission, Assessment, Residency, Selection, Specialist medical training, Surgery, Surgical education, Surgical training, Trainee

## Abstract

**Background:**

There is an increasing global interest in selection processes for candidates to surgical training. The aim of the present study is to compare selection processes to specialist surgeon training in the European Union (EU). A secondary goal is to provide guidance for evidence-based methods by a proposed minimum standard that would align countries within the EU.

**Methods:**

Publications and grey literature describing selection strategies were sought. Correspondence with Union Européenne des Médecins Specialists (UEMS) Section of Surgery delegates was undertaken to solicit current information on national selection processes. Content analysis of 13 semi-structured interviews with experienced Swedish surgeons on the selection process. Two field trips to Ireland, a country with a centralized selection process were conducted. Based on collated information typical cases of selection in a centralized and decentralized setting, Ireland and Sweden, are described and compared.

**Results:**

A multitude of methods for selection to surgical training programs were documented in the 27 investigated countries, ranging from locally run processes with unstructured interviews to national systems for selection of trainees with elaborate structured interviews, and non-technical and technical skills assessments. Associated with the difference between centralized and decentralized selection systems is whether surgical training is primarily governed by an employment or educational logic. Ireland had the most centralized and elaborate system, conducting a double selection process using evidence-based methods along an educational logic. On the opposite end of the scale Sweden has a decentralized, local selection process with a paucity of evidence-based methods, no national guidelines and operates along an employment logic, and Spain that rely solely on examination tests to rank candidates.

**Conclusion:**

The studied European countries all have different processes for selection of surgical trainees and the use of evidence-based methods for selection is variable despite similar educational systems. Selection in decentralized systems is currently often conducted non-transparent and subjectively. A suggested improvement towards an evidence-based framework for selection applicable in centralized and decentralized systems as well as educational and employer logics is suggested.

**Supplementary Information:**

The online version contains supplementary material available at 10.1186/s12909-021-02779-5.

## Introduction

The selection of candidates to surgical training has been called a “missing link” in surgical patient safety work [1]. Regardless of how good the education and training system is, selection of candidates to undergo education and training is crucial. An important question is thus if the right candidates are trained to become future surgeons. Currently, there is a lack of evidence on which instruments can predict future competence [2]. However, there is an increased global interest in evidence-based recruitment and selection processes for specialist surgical training [3–6].

Assessment tools for selection have mainly been investigated in Anglo-Saxon countries such as Australia, Canada, USA, New Zealand, Ireland and the UK concomitant with profound reforms of the recruitment and selection processes to improve surgical training [2]. Most of these countries use centralized systems for selection. This is combined with structured interviews, psychological and aptitude tests based on national templates with exact grading according to specific criteria to predict clinical performance [7–9]. In contrast some countries such as Sweden apply a traditional, locally-based, decentralized system for selection to surgical trainee positions without formal guidelines or firm selection criteria [10].

The purpose of this report is to delineate current selection procedures in EU member-states and identify salient bases for differentiating these. Cognizant of the heterogeneity that also exists within centralized and decentralized systems, the advantages and disadvantages of these two paradigmatic systems are discussed. Avenues for improving selection processes with evidence-based tools independent of the underlying structure are suggested.

## Material and methods

Information on bodies in EU countries responsible for surgical training programs and assessment tools for selection were obtained through Google searches and PubMed. A string of keywords was used including name of country, “surgical”, “trainee”, “selection”, “specialization” or “application.” Websites and “grey literature” [11] on the application and selection processes to surgical training were identified. Subsequently, a letter was sent to 16 Board Delegates in the Section of Surgery of the Union Européenne des Médecins Specialists (UEMS) to obtain information concerning the respective country’s selection process. One reminder was sent to non-responders. The data for this article derives from an ongoing multi-disciplinary intervention project on recruitment and selection processes to specialist surgical training in Sweden. All data pertaining to human subjects was obtained in adherence with relevant guidelines and regulations for the wider project’s ethical clearance obtained from the Swedish Ethical Review Authority (case number 2016–1050). The application and selection processes were sorted into three categories: centralized selection was defined as having a single national application, selection, and admission/employment process; hybrid as having one or two of the three; decentralized selection process as having none of the above undertaken at the national level.

### Comparative case study of a decentralized (Sweden) and centralized (Ireland) process for trainee selection

Flyvebjerg argues [12] comparative case studies provide deeper and more sophisticated insight into complex, context-dependent phenomena than searching for context-independent, aggregate-based generalizations (rules). Sweden was selected as an archetypical case of a decentralized selection system. Data on selection procedures in Sweden was obtained via available literature [10] together with semi-structured interviews with 13 experienced surgeons, as previously described [13] and knowledge within the research group. Oral informed consent as per Swedish Ethical Review Authority (case number 2016–1050) was obtained from all informants, who were informed of the purpose of the study, that their contributions would be analysed and can come to comprise part of published results of the study, and that they would not be cited by name in any public documents. Permission to record interviews was obtained at the opening of all recorded interviews. One interviewee did not consent to be recorded and notes were taken in this interview. The theme of selection emerged in the interview material and served as a starting point for the current study.

Ireland was selected as a polar opposite case to Sweden, being the most centralized and formalized system for selection of surgical trainees in the EU. To obtain comparable insight, the research team conducted two study-visits to the Royal College of Surgery Ireland (RCSI) to observe the current testing and selection procedures. Visit 1 (2018) consisted of observation of interviews and selection of candidates to Core Surgical Training. Visit 2 (2019) consisted of observation of the interviewing and selection of candidates for the cut to Higher Surgical Training which was performed after the 2 years of basic training.

During the visits, relevant material describing the selection process was shared by RCSI faculty and staff, fieldnotes were taken during observation of the selection process and combined with spontaneous interviews with faculty members and involved staff [14]. Fieldnotes were pooled after each of the two visits to the RCSI and analysed by a team of two sociologists and four surgeons [14]. Focus during the study-visits was on the operationalization of the selection instruments and procedure, discussions of intended and unintended effects, challenges and benefits of the selection system in general and specific instruments, and the preconditions for their utilization. Data from the site visits and supporting documents provided the core background for Ireland as archetypical of the centralized system is based.

## Results

### Mapping

**Figure 1:**
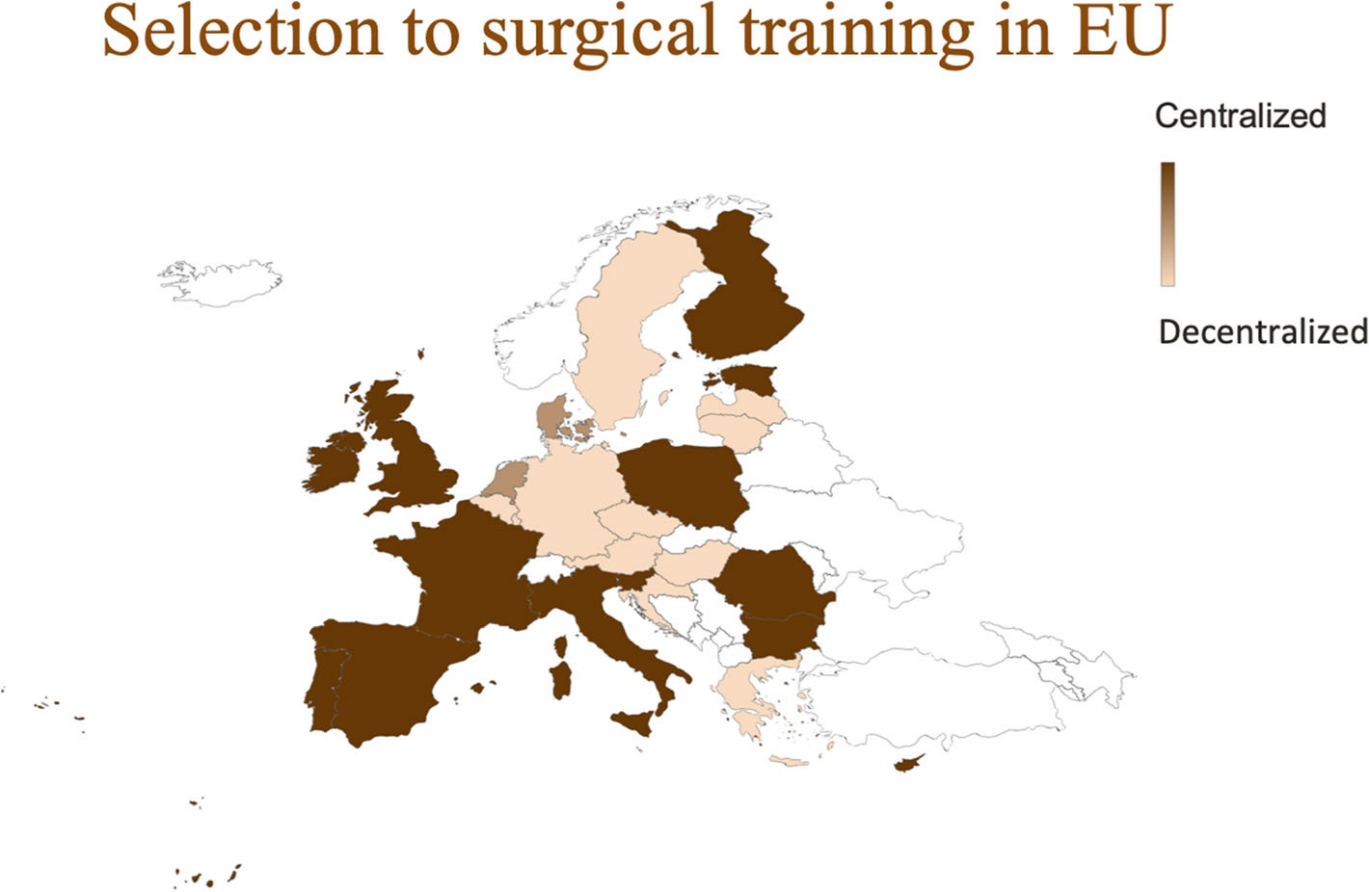
**Fig. 1** The selection process to surgical training programs in the EU is organized in a centralized, hybrid or decentralized fashion. See Table 1 for explanations on the associated educational or employment logic

Selection processes in 27 countries were analysed and divided into three types of selection on a scale from centralized to hybrid to decentralized (Fig. 1, Table 1). Slovakia was excluded due to lack of information in English. Answers from seven UEMS Board delegates were obtained (Additional file 1). A wide range of selection processes using different assessment tools were documented, from a strict national and centralized process to a completely decentralized system down to the local hospital level. Only a few countries conducted transparent, standardized selection with clear statements of the weight of each criterion. Some countries have standardized selection, but with no clear statements on the weighting of criteria, while other countries, like Sweden, have no official standardization or guidelines for selection of trainees.

**Table 1:** Selection processes to surgical training programs in EU (refences in Additional file 2)

Country	Announcement of positions	Selection process	Decision authority	Selection control
**Austria (1–3)**	Hospital Publicly advertised tenders are open all year round, based on the needs and availability in various hospitals or medical centers.	Direct application for the desired residency program to the chosen hospital. Traditional job application and interview, Motivation letter, CV, Copy of degree. Head of department decide after interview.	Local Hospital	**Decentralized**
**Belgium** (personal communication, Oct 2, 2017) (4)	University Local Application to University Hospital Intake once/year	1. Send cv and cover letter to dean of faculty of medicine 2. Apply for & pass selection test Undergraduate medical education entry test “ test d’orientation “. 3. Local recruitment in seven medical faculties, however in a similar way. Selection based on results from medical school, results from surgical internship and research equals 75% and the interview 25%.	Local 7 university Hospitals	
**Croatia (5–7)**	Hospital Local public tenders published by different	The selection of the resident to the specialization is determined by an interview and based on a number of criteria (interview score, grade average, participation in additional training or publications during and post-graduation, etc.)	Local If approved by the Ministry the applicants can choose the institution where they would like to practice the training.	
**Czech Republic** (personal communication, Oct 5, 2017) (8)	Hospital Local advertisement	Traditional job application and interview, Motivation letter, CV, Copy of degree. Head of department decide after interview. Due to lack of applicants almost everyone gets accepted/ Lack of applicants leading to “almost no selection criteria”.	Local Hospital	
**Germany (9, 10)**	Hospital Local/Individual advertisement at hospital website	Local application directives. Traditional job application and interview; Motivation letter, CV, Copy of degree. Head of department decide after interview.	Local Hospital	
**Greece (11, 12)**	Hospital Regional advertisement at local authority for public health website	There are no specific application deadlines for applying for a training position. The procedure works on a “first come, first served” basis, that means that priority is given to an individual only according to the date of submission of their applications	Local Hospital	
**Hungary (13, 14)**	National annual decision on regional needs. University application (4 universities)	Applicants are ranked according to points, which are comprised of the following: average of the comprehensive examination results and the exam results of specific profession-specific subjects (as semester points), the various scientific and language examination achievements (as additional points), and the points determined over the course of the personal interview. The University may repeat its call for applications to fill the unfilled positions.	University	
**Latvia (15)**	University application yearly	Application for maximum 2 specialties, ranking order. Motivation letter, recommendations, diploma, scientific work in last 7 years, certificates for PGT, CV and an interview are evaluated.	University	
**Lithuania (16, 17)**	Local University Once yearly	Selection via one of the two universities according to a calculated score.	University	
**Luxembourg** (personal communication, Oct 20, 2017)	Local Hospital	Based on recommendations from university and interview.	Local Hospital	
**Sweden (18)**	Local Locum position or Individual advertisement at hospital website	See Table 2 for details.	Local Hospital	
**Denmark (19–21)**	Hybrid advertisement	Regional application for main trainee years in one of three regions. The regions have different selection processes where some use structured interviews and some Multiple Mini Interviews. Interview is mostly weighted as selection criteria.	Regional Hospital	**Intermediate**
**Netherlands** (personal communication, Oct 3, 2017)	National	Applicants choose to apply for two of eight regions. The regions program directors paper select which candidates to interview. No formal guidelines for selection. By tradition: Faculty interview or two separate interviews (multiple). Clinical performance, scientific achievements, social involvement, sport or music is valued.	Hospital Regional	
**Bulgaria (22)**	National University Application	Obligatory examination process.	University	**Centralized**
**Cyprus (23)**	National Once yearly	Written examination, one for surgical specialties and one for non-surgical. For those succeeding, an oral examination follows.	Accredited hospitals, by Ministry of health in Greece	
**Estonia (24)**	University/national Once yearly	Obligatory entrance examination.	University	
**Finland (25, 26)**	Application via university Application periods 2/year	Central Maximum 3 specialities or same speciality at 3 different universities. Clear criteria statement in application advertisement. A maximum of 20 points can be obtained (Work experience (10p), scientific experience (8p) and priority (2p)) Structured interviews by at least 2 interviewees is conducted with 2–3 applicants per position.	University	
**France (27, 28)**	National Once yearly	National ranking examination after which the students nominate their specialty of interest and geographical considerations. National matching process based on the previous.	University	
**Ireland (29, 30)**	National Once yearly	See Table 2 for details.	University	
**Italy (31)**	National Once yearly	Obligatory entrance examination A single national ranking based on the exam.	University	
**Malta** (personal communication, Oct 22, 2017) (32)	National	Only one teaching hospital for surgical training. Selection based on a structured interview and research is given extra points.	Hospital	
**Poland** (personal communication, Oct 24, 2017) (33)	National	National guidelines for selection, 90% weighing from a national post-graduation exam and 10% from an interview. Due to few applicants, everyone gets accepted.	University	
**Portugal (34–36)**	National Once yearly	Examination will rank you at the national level according to your exam mark (80%) and your medical school grade (20%), and thus your position in the competition for training posts. After the exam, the candidates start their foundation year in January. Since 2016, the available training positions are not enough for all the candidates.	University	
**Romania (37)**	National	National exam leading to ranking of applicants who then choose their preferred specialisation.	University/ University accredited Hospitals	
**Slovenia (38)**	National advertising twice a year	Public advertising twice a year from the Medical Chamber of Slovenia. Interview committee; National coordinator, Medical Chamber representative, Representative from region. Selection criteria: interview scores (35 p), University average grade (35 p), letters of recommendation (30p).	Accredited Hospitals. Payment by national healthcare insurance company or self-funded	
**Spain (39)** (personal communication, Oct 19, 2017)	National	Ranking of candidates based on: Knowledge test weights 90% Academic grade medical school 10% Highest rank get to choose speciality and location first.	Accredited Hospitals	
**UK (40–43)**	National advertisement	The intake to the 2 year common Foundation programme is co-ordinated by its own institution that is shared between the agencies in England, Scotland, Wales and northern Ireland: United Kingdom Foundation Programme Office. For selection to Core Surgical Training: a structured interview process, including a formal application, preparation of a portfolio of surgical and medical experience, and progression through a number of structured interview stations. Extensive guidelines exists. Portfolio 34% Management 33% Clinical 33% All eligible are guaranteed interview, which is conducted in three parts; management station, portfolio station and clinical scenario station. There is a cut-off score for the summarized interview score and also a possibility of receiving “unsuccessful ticks” during the interview, those below the cut-off or with too many unsuccessful ticks will not be ranked. From the successful candidates there will be a national ranking and the applicants choose their preferred program.	National	

Excluded: Slovakia

Interviews were found to be the most frequently used assessment tool, but type of interview ranged from ad hoc to structured Multiple Mini Interviews (MMI). The use of evidence-based assessment tools in the selection process also varies, from combinations of a multitude of validated tools to only test results or an interview (Table 1).

Twelve countries have decentralized selection processes with recruitment, evaluation and selection decision-making by local universities and medical schools or hospitals. Two countries have hybrid systems and 14 countries have national centralized systems. None of the processes are identical across countries, and the elaborateness of the centralized systems vary considerably.

Associated with the centralization-decentralization scale which constitutes a significant difference among countries is whether surgical training is primarily governed by an employment or educational logic. In some countries where a college or university is the responsible organizing entity, the applicant to a trainee position is considered a “student”, and a centralized admissions or selection process similar to other higher studies is performed. This is exemplified by the Irish case. Other systems, usually decentralized, where hospitals select candidates to surgical training, and do so as immediate or future employers, act according to an employment logic.

Ireland has the most elaborate centralized selection process, and the only country formally testing technical skills and aptitude. Spain has a centralized recruitment process where written exam scores are translated into a rank and hospital positions are based on this rank only. In other countries, for example Poland with a national selection process and guidelines for selection, there are fewer applicants than positions, meaning that more or less all applicants are accepted (written communication UEMS delegate, Oct 24, 2017) (Additional file 1). Some countries such as Estonia and Malta have de facto centralized systems due to country size, with only one teaching hospital.

Denmark is an example of a hybrid centralized-decentralized system, consisting of a national application system whereafter regional healthcare organizations perform the final selection of candidates. Likewise, in the Netherlands, after applying through a centralized national system, candidates choose the regions they wish to work in and varied regional selection procedures ensue. Finland changed its system during the study period, from a locally run and less structured process to a centralized system with selection through the universities with structured interviews and clear criteria for evaluation.

Belgium represents a country with decentralized local selection but a standardized consensus selection process across each of the seven university hospitals (Additional file 1). In Greece, training positions only exist in public hospitals and are generally advertised on the official website of respective local authority for Public Health. There are no specific application deadlines for applying for a training position and selection process works on a “first come, first served” basis, i.e. priority is given to an individual only according to the date of submission of their applications.

### Comparative case study of two representatives of archetypes: Sweden and Ireland

Comparison of the decentralized Swedish and centralized Irish selection processes is visualized in Table 2. Sweden conducts strictly local selection processes at each hospital without standardized guidelines or consensus on selection criteria. An informal system exists with a 6–12 month locum position in the surgical department for vacant or temporary positions. The head of department and program director then decides whether or not a permanent position as a surgical trainee is offered based on employment needs. The lack of transparency and the unstructured process of assessment during the locum was considered problematic by the interviewed surgeons. However, support for the locum system was quite strong as it was deemed appropriate to evaluate a candidate’s fit for the job. A common theme for those not entirely satisfied with the current system concerned the need to apply structured and validated assessment tools as they had experienced unsuitable candidates slipping through the system [13]. A trainee usually receives a permanent position in the department when training is completed. This was stated as the main reason why the surgeons felt concerned about unsuitable surgical trainees in order to avoid a dysfunctional, ill-suited colleague.

**Table 2:** Comparison of selection process in a centralized vs decentralized country

		**Centralized (Ireland)**	**Decentralized (Sweden)**
Governing body responsible for training		Royal College of Surgeons UNIVERSITY based education	Local Hospital APPRENTICESHIP model. Guidelines from Swedish surgical Society and National Board of Health and Welfare
Announcement of training positions		Core Surgical Training positions are announced once/year and open to all eligible applicants.	Local traditions at each recruiting hospital. Locum positions are customarily not externally announced.
**Application process**			
	Checklist of eligibility, required documents and exams	Checklist available	No standardization or checklist exist Trainee positions are internally and/or externally announced depending on employer’s choice.
	Personal letter	-	Email or letter to head of surgical department or head surgeon responsible for trainee doctors
	CV	Standardized. 1) Verification of presentations, publications 2) case reports 3) audits 4) attendance at meetings, courses	Not standardized No checklist available
	Reference forms	Two references required	Not standardized Contact details to one or two references
	Letter of support from supervisor	If applicable	-
	Exams	1. MRCS Part A/B results If applicable 2. Verification of Eligibility for the Irish Medical Council Trainee Division	Medical exam and passed internship resulting in medical license.
**Assessment of candidates**			
Tool	IRELAND		SWEDEN
Exams	Undergraduate academic record 15% of total score	Objective assessment	Pass/fail medical exam
Aptitude	Surgical Aptitude 15% of total score	Psychomotor skills, Visuospatial ability perception Laparoscopy test	Not investigated
Interview	Multiple Mini Mini Interviews at four stations, each lasting 8 min, 2 faculty members interview and rate individually		No guidelines exist. All interviews differ between hospitals and depend on the head of departments own structure. Clinical scenarios are not customarily assessed.
	Clinical judgment 15% of total score	Structured sequential clinical scenario × 2	
	Interpersonal skills 15% of total score	Communication Teamwork/Leadership Crisis management Negotiation/conflict resolution	
	Professional Development 15% of total score	Clinical research projects Attendance at meetings /courses Audit projects Teaching activities	
	Suitability for speciality training 15% of total score	References Motivation/drive Knowledge of speciality Time/stress management Work ethic/professionalism	
**Score & ranking**	Ranking of candidates. Highest score chose first	Maximum score 100	Not performed in a structured and transparent manner
**Cut**	After 2 years selection for higher surgical training is conducted		-

In Ireland, the selection process is well-described on the RCSI website for potential candidates [15]. Ireland’s surgical training takes place in two steps with a selection process for each. Selection for Core Surgical Training is included in Table 2. After 2 years a second selection is made to Higher Surgical Training. During the first selection, around 100 candidates compete for 60 positions, and candidates are tested for technical and non-technical skills. After technical testing, a session of four consecutive eight-minutes Multiple Mini Interviews (MMIs) with two assessors from different surgical specialties are conducted. The four stations focus on different professional areas and the assessors independently electronically rate the candidates on a Likert scale. The members of the faculty were aligned through a common information meeting in the morning before the interview and testing process started. Instructions and rules for how to conduct and document the MMI assessment were provided. Furthermore, the Department for Quality Assurance at RCSI monitors the process in real time to assure alignment in judgement of the assessors during the MMI sessions. Personal knowledge of a candidate was not an exclusion criterion for being an assessor.

The RCSI has developed a metricized system for observations during the MMI and assessments of academic records and aptitude tests to rank the candidates. The highest ranked candidate chooses hospital and training position first. The metrics from the first selection process are adjusted to a maximum of 100 points. A majority of the assessors were satisfied with the process, and support for the system is strong, though a few faculty members mentioned concerns about the lack of possibility of identifying unsuitable candidates per se during the short interviews with standardized questions.

After 2 years of training, with a variety of compulsory academic, pedagogical and practical activities, a selection takes place to Higher Surgical Training. For this round of selection, a maximum of 1000 points could be collected through the above-described activities and another round of MMIs, where the interviews comprised 400 points of the total score. Some candidates missed the final selection by less than 10 out of 1000 points [16].

After scores are compiled, the assessors were able to discuss the candidates and scores but are not allowed to make adjustments to the final result, even in cases where they were frustrated over exclusion of a candidate they found very promising by a slim margin. The lack of possibility to directly impact the choice of a future colleague was clear and some faculty members stated that the only way to have any impact was to participate in the process. This, along with a collegial sense of duty ensures a sufficient number of quality assessors to maintain the very personnel-intensive system. A further systemic benefit according to the RCSI program director is that the attrition rate has been reduced substantially after this rigorous process for selection has been applied in tandem with follow-up on skills during the first two trainee years.

## Discussion

This study shows great variations in how surgical trainees are selected and in the application of validated assessment tools.

A comparison of centralized and decentralized systems, especially the specific cases of Sweden and Ireland, shows particular advantages and disadvantages associated with each selection system. The Irish centralized system benefits from running an annual and open competitive national recruitment process that increases the number of candidates to choose from. The selection process is systematic, transparent and concentrated, and all candidates are compared in line with the BEME (best evidence medical education) guide no 45 [2]. A centralized process with a high degree of standardization mitigates the risk of decisive individual bias and secures a transparent and equal process for all applicants [17].

A further advantage of the current centralized standardized system in Ireland is that it reportedly lowered the attrition rate in the training programs (Prof. O.Traynor, Dean Of Postgraduate Surgical Education &Training, personal communication, March 20, 2019). A low attrition indicates efficacy of the instruments used for assessment, i.e., how well they test for capabilities needed for completing the educational program. Declining attrition rates also have clear economic benefits. However, it is difficult to compare attrition rates across centralized and decentralized selection systems, due to the lack of data in decentralized systems. The case in point: in Sweden the total number of surgical trainees at any given time is not recorded. Keeping track of the trainees over the course of their training allows for evaluation of cost, attrition, and effectiveness of the training program in each hospital. This is more feasible and practiced in centralized systems. However, it is not impossible to implement in a decentralized context if drop-outs are reported to a central entity charged with collecting and analysing such data.

Centralized systems for selection with systematic assessment tools are also the ones producing knowledge on the subject of selection with sufficient volumes of observations in controlled contexts, as shown by the Anglo-Saxon countries [5, 6, 18–20]. Extensive selection systems receive more attention, resources and scrutiny devoted to their operation, maintenance and improvement. Likewise, the more centralized the system, the more transparency and accountability is required for legitimacy. On the other hand, more sporadically used systems implemented on fewer numbers of candidates and involving fewer individuals in the recruitment evaluation and selection process, garner less attention, critical assessment and produce less documentation. Thus, centralized systems more frequently produce evidence-based data on instruments and procedures, data that can also be utilized by decentralized systems. This is reflected in that almost all studies on assessment tools for selection were conducted in the centralized Anglo-Saxon contexts [2, 21].

A central virtue of local or decentralized systems is flexibility, responsiveness and adaptation to local needs [22]. Depending on how processes are implemented the flexibility of decentralized systems can be advantageous or disadvantageous.

Recruitment from the locum system was deemed by the experienced Swedish surgeons to be positive as it allowed decisions to be made after observing the candidates´ work in situ [23]. Previous job performance in the department is considered a valid way to assess a candidate if done in a structured way by peer ratings [24–26]. Such a system allows for a holistic assessment over time compared with a “collection of indicators” rendered through a series of discrete assessments produced by multi-instrument centralized systems. This decentralized selection process benefits from being flexible and allows for locum positions whenever need arise, e. g. parental leave, turn-over or health issues. It also increases the control over the process for the local head of department and allows young doctors to try several different specialties. On the other hand, a disadvantage is, as the case for Sweden, that there is currently no national consensus on assessment methods for selection of candidates to surgical training programs.

The decentralized and unstandardized processes can lead to happenstance playing a significant role in the selection process with locals or “insiders” having undue advantage especially if the trainee positions are not announced externally. Also, having more than one candidate to choose from affords comparative evaluative opportunities and reduces happenstance playing a role.

Most selection systems are oriented towards finding the top candidates, but an important consideration when selecting to training positions is also to identify *unsuitable candidates* to mitigate the multiple detrimental effects of enrolling a potentially problematic trainee and future colleague [13, 27]. Trainees that need remediation will inevitably affect patient safety, work climate, and are costly for the department [28–31]. On the one hand, local selection processes are particularly susceptible in that they de facto narrow the candidate field with an increased risk for selecting a person with a slow or dysfunctional learning curve or hazardous traits [13, 27]. On the other hand, prolonged exposure of a candidate to multiple senior colleagues and members of other professions in a variety of settings would result in deeper familiarity and offer greater opportunities to detect both unsuitability and fitness for the job [13, 32]. Hazardous traits may be detected through validated tools or clinical job performance if appropriate attention is paid, albeit currently not performed to the best of the authors knowledge [13, 27, 33, 34].

As shown in Table 1, a majority of the countries rely on academic examination (six) or non-MMI job interviews (ten) in their selection process. These methods have relatively low predictivity for work performance according to literatures in industrial organizational psychology and current evidence in post-graduate medical education [2, 6, 25]. As pointed out in BEME guide no 45, test scores and grades are insufficient in selecting applicants for a complex and multidimensional role like specialty training. Likewise, traditional CVs and letter of recommendations are inconsistent in predicting performance [2, 6].

Regardless of having a centralized, decentralized or hybrid selection system, the interview is the key assessment tool. The current study indicates that structured interviews are not often used, although they are considered to be one of the most reliable tools for assessment with twice to three times the predictive value compared with unstructured interviews [25, 35, 36]. Even with only a handful of applicants for each position, MMIs have also been shown to be reliable and well suited to evaluate non-cognitive skills [19, 37, 38]. However, conducting MMIs may be too resource-demanding in decentralized systems with few applicants [39], whereas the cost of implementing a structured interview would be reasonable and advantageous. Structured interviews allow probing for non-technical skills, warning signs for unsuitability and simultaneously finding top achievers [40].

With the current pandemic of Covid-19 there are new challenges arising when interviewing candidates. The transition to video-mediated communication (VMC – Skype / Zoom) recruitment interviewing instead of face-to-face (FTF) interviews can save time and decrease cost for the applicant, but concerns have been expressed if there are disadvantages [41]. Research has been conducted for over a decade on VMC versus FTF interviewing and early studies of those actively engaged in interactive interviewing tend to show little difference between FTF and VMC on the impressions of assessors of job candidates [42–44]. However, a recent study found significant differences in assessments of passive assessors, i.e. those not actively participating in the conversation, but watching it, indicating that there are varying impacts on assessors depending on their roles in interviewing [45].

Although alluring to surgeons, evidence on selection using assessment instruments and procedures specific for surgical training, for instance assessing technical aptitude and visuospatial ability, has yet to be proven. In Ireland aptitude testing is conducted only for the first round of selection, i.e., for core surgical training [4, 46–48]. Aptitude testing is costly and resource demanding. Its importance and optimal weighting between different assessment instruments is unknown, as is correlation to later surgical performance.

Situational judgment tests (SJTs) have been found to reflect job performance and tend to discriminate accurately between average and low performing participants [49, 50]. Situational judgment tests could be used as an initial screening method before conducting more costly and time-consuming assessments, especially in situations with a low applicant to vacancy ratio [19, 20, 51]. Other assessment tools that have been investigated as suggested screening tools for successful residents are personality testing, emotional intelligence (EI) and grit, but without proving a clear benefit like the SJTs in distinguishing between candidates at the level of selection [52, 53]. Grit is interesting since it has been found to be higher amongst consultants than trainees, and proposed to be associated with attrition amongst residents, but only few and underpowered studies exist and there is a lack of large and longitudinal studies [54–56].

Surgery is a high-stakes occupation. Insufficient critical attention to selection, conserved traditional practices, and reluctance to use decision aids and systematic procedures is not unique to this field [35]. However, the consequences are uniquely inappropriate due to high risks to patient safety and work environment. Current practice in most countries is sub-optimal with room for improvement in both centralized and decentralized systems.

Many centralized systems would benefit from utilizing more validated instruments, especially combinations thereof, and have volumes of applicants to feasibly do so. Decentralized systems can benefit from the collective and cumulative learning of developing and deploying more standardized systems across local units. Standardization is not the same as centralization. In countries where a hospital is the organizing entity for recruitment and selection of surgical trainees, there is often an employment logic, i.e., the applicant considered a part of the workforce, and thus selection for the job is conducted like any other apprentice position. In contexts where surgical training is associated with more or less permanent employment at the unit where training takes place, transition to a centralized system is unlikely. Even in such contexts, a standardized selection procedure preferably composed of validated or evidence-based components, and sharing data across like units, is possible to develop and would be beneficial [57].

### Limitations

There are several limitations in our study. The countries selected were limited by language and responding UEMS surgical board members, leading to a possible selection bias. Despite these differences, the material was considered valuable in describing the overall features for selection of trainees to surgical programs across the EU in a comparative manner. A limitation is that researchers were only able to study two prototypical examples of a centralized and decentralized system in depth.

A strength of the conducted study is that the research group consisted of social scientists and surgeons, which allowed for researcher triangulation and contributions on qualitative research competence, human resource management competence and the experience of heads of department with personnel and hiring responsibilities in Sweden. Another strength is that data was collected over an extended period of time, which gave the opportunity for iterative data collection and discussion within the group.

## Conclusion

A wide variety of selection processes to surgical training programs and limited use of evidence-based assessment tools were found across the EU. Comparing systems reveals advantages and disadvantages of different selection systems with modern evidence-based processes found in reformed centralized rather than traditional systems. Based on these findings and current evidence, recruitment and selection to surgical training in many European countries has great room for improvement.

A standardized procedure composed of validated evidence-based components could be developed and distributed across decentralized units as well as a fixture of centralized systems, independent of employment or educational logics without increasing cost significantly.

A selection framework adopting current evidence-based instruments would include 1) ensuring multiple candidates to select from per position through wide advertisement and recruitment efforts; 2) conducting multiple mini interviews (MMI) or structured interviews by at least two assessors; 3) applying situational judgment testing (SJT); 4) conduct structured validated assessments during job trial periods with SJTs (if trial work periods are employed). An unresolved issue is that the optimal weighting between these different instruments. Likewise, even though surgery is a technical craft, the evidence is still equivocal concerning technical skill and aptitude assessment at the point of selection.

## Supplementary Information

**Additional file 1.** Letter sent to 16 members of the UEMS Board of surgery with a request for detailed information in how the country were selecting their surgical trainees.

**Additional file 2: Table 1**. references.

## Data Availability

All data concerning selection process generated or analyzed during this study are included in this published article and its supplementary information files. The datasets used and/or analyzed during the current study are available from the corresponding author on reasonable request (interview and observation data are not available for release beyond the research group as per ethical clearance stipulations to protect the respondent’s anonymity).

## Abbreviations

EU: European Union
BEME: Best Evidence Medical Education
MMI: Multiple Mini Interviews
RCSI: Royal College of Surgeons Ireland
SJT: Situational Judgment Test
UEMS: Union Européenne des Médecins Specialists
