# Supplementary material for: Different approaches to selection of surgical trainees in the European Union
Source: BMC Med Educ. 2021 Jun 30;21:363. doi: 10.1186/s12909-021-02779-5 (PMC8243060; doi:10.1186/s12909-021-02779-5)
Supplement: Supplementary file 1 — Additional file 1. Letter sent to 16 members of the UEMS Board of surgery with a request for detailed information in how the country were selecting their surgical trainees. [file 12909_2021_2779_MOESM1_ESM.docx]

**Different approaches to selection of surgical trainees in the European Union**

Kristine Hagelsteen^1,2^, Hanne Pedersen^2^, Anders Bergenfelz^1,2^, Chris Mathieu^3^

^1^Lund University, Department of Clinical Sciences, Surgery, Lund, Sweden

^2^Practicum Clinical Skills Centres, Skåne University Hospital, Lund, Sweden

^3^Lund University, Faculty of Social Sciences, Department of Sociology, Lund University, Lund, Sweden

**Appendix A**. ***Letter sent to 16 members of the UEMS Board of surgery with a request for detailed information in how the country were selecting their surgical trainees.***

**Questions asked**
Is the process run local or centralized?
Which methods are used during the selection process? 
Which criterions are mostly weighted? 
Are there any cuts during the training years?                    
If you have any available guidelines on the selection process we would appreciate them in order to compare the differences in detail.

**Answers from responders in alphabetical order**

**Belgium**

It is mandatory in Belgium that the selection is driven by the universities. There are seven medical faculties (four on the Flemish part and three in the French-speaking part), each organizing its own selection during the last year of medical studies (6 years). There are probably some small variations from one university to the other but roughly it is the same pattern: is taken in account the medical studies results of years 4, 5 and eventually 6 (in case of candidates ex-æquo), the results of internship in surgery done in the university setting (at least two months), participation in a research (clinical or laboratory). This will represent 75% of the points and for those who can make it, there will be an interview with a panel of members of the University surgical department and external consultants ( this will represent the last 25%).

During the interview, the evaluation is not on theory but more on motivation, technical and non technical skills, behavior in certain situation...

This will give a classified list of candidates. The number of candidates allowed to start their six years training will depend on the number of posts available in each university; this is discussed between the deans of each universities (french on one side and flemish on the other but with no contact in-between them).

The candidates have to rotate between Universities and non-universities centers (a minimum of two years in each), changing very year or every two years. They have to fill a on-line log-book with all the procedures they are doing ( assisting and first-hand) and this is analyzed every year by a Chamber of recognition (one on each side of the country) who checks the quality of the training and exposure. They have to do one oral presentation in an international meeting and write down a paper in an international review with a selecting committee.

I think I have given you a fair view of the way it works in Belgium, though I forgot to tell you the trainees have to undergo an exam after their second year (basic science) and one final exam on the sixth year (OSCE scheme)...

Thanks for your comments.

Actually, the interviews are probably different from one university to another. Participating at two of them on the French speaking side, they are very much in the same frame: it is more a discussion about motivation, vision of their profession in the future, creation of decision-making situations, ... It lasts about 15 minutes. There are never theoretical questions, since they are supposed to have succeed their exams.

Good luck...

By the way, we are seriously thinking of introducing an UEMS exam may be after second year on basic science ( instead of the national exam ) and may be in the final year a General Surgery exam. But it will take time before everybody around the table agrees

**Czech Republic**

Unfortunately I have to state that in the Czech Republic there is such a lack of surgeons ( or students interested in surgery ) that local hospitals ( even an University Departments ) do not use almost any selection criteria.

**Luxembourg**

As we don't have University in our country, the process for selection starts already outside from Luxembourg. Those who are chose for trainee are coming to us on recommendation from the University.

We see them and approve on recommendations and interview.

**Malta**

It seems most countries practice a combination of grades from Medical school, CV, references and interviews. How is the practice in your country?

A call is issued and any doctor who has finished two years foundation course can apply. They are selected by a structured interview.

Is the process run local or centralized?

We have only one teaching hospital in Malta

Which methods are used during the selection process?

Interview

Which criterions are mostly weighted?

Research courses publications are given extra points

Are there any cuts during the training years?

Yes. If a trainee does not pass exams or assessments he has to repeat. If the trainee does not pass within 10 years he cannot continue. 
If you have any available guidelines on the selection process we would appreciate them in order to compare the differences in detail.

I am copying in my secretary and asking her to send you a Basic Specialist Trainee interview form.

**Netherlands**

Is the process run local or centralized?
All candidats have to register centrally and can choose 2 of 8 regions to apply. The regions (program directors of the hospitals (6-8) form a region) do a paper selection of the applications and select which candidates they want to interview (mostly half of which 30-50% will be recruited)
Which methods are used during the selection process? 
That differs between regions, some have a panel of program directors that conduct the interview, some (as we have) make 2 smaller committees and have 2 separate interviews and afterwards discussion who were the best candidates
Which criterions are mostly weighted? 
Excellent clinical performance by reference is essential, significant scientific achievements, social involvement (student committess, socials skills) and sport or music are important
Are there any cuts during the training years? 
Prior clinical experience as house officer can lead to reduction (1/2-1 year max) of the training period prohibited that entrusted professional activities have been granted

If you have any available guidelines on the selection process we would appreciate them in order to compare the differences in detail.

There are no real formal guidelines. The regional committees are relatively free in conducting the selection process as they want and so far satisfactory

**Poland**
Selection criteria in Poland are similar for all medical specialities and are regulated by government.

90% od weight is result of national post graduation exam and 10% is interview.

Current practice is less optimistic. There are less applicants than places for trainees in surgery, so everyone who applies is accepted.

Casual training program takes 5 years followed by national exam.

**Spain**

1.- national examination, 250 question exam (75%) plus 25% cv.

The examination works for all the different medical specialties. Usually > 6000 applicant. Common national list and applicant choose center according the list number

2.- national  single day simultenous common exam

3.- examination outcome plus medical school results

4.- no cuts during the yrs. continous monitoring by resident tutor

5.-
