# Supplementary material for: Different approaches to selection of surgical trainees in the European Union
Source: BMC Med Educ. 2021 Jun 30;21:363. doi: 10.1186/s12909-021-02779-5 (PMC8243060; doi:10.1186/s12909-021-02779-5)
Supplement: Supplementary file 2 — Additional file 2: Table 1. references. [file 12909_2021_2779_MOESM2_ESM.docx]

**Different approaches to selection of surgical trainees in the European Union**

Kristine Hagelsteen^1,2^, Hanne Pedersen^2^, Anders Bergenfelz^1,2^, Chris Mathieu^3^

^1^Lund University, Department of Clinical Sciences, Surgery, Lund, Sweden

^2^Practicum Clinical Skills Centres, Skåne University Hospital, Lund, Sweden

^3^Lund University, Faculty of Social Sciences, Department of Sociology, Lund University, Lund, Sweden

**Appendix B. Table 1 references with numbering according to country.**

| Country | References |
| --- | --- |
| Austria | 1. Knöbel A. How to apply for a medical or surgical specialty training post in Austria with a UK/Foreign medical degree [cited 2021 Jan 5]. Available from: <https://anjaniknobel.com/2017/04/29/doctors-how-to-apply-for-specialty-training-in-europe/>  2. Public Health in Austria: EURONET MRPH; [Available from: <http://euronetmrph.org/residency-austria/>.  3. MEDICALMOBILITY. Austria: MEDICALMOBILITY.EU; [cited 2021 Jan 5]. Available from: <https://www.medicalmobility.eu/austria>. |
| Belgium | Personal communication, Oct 2, 2017  4. Jouini C. Belgium: Medical Residency Database; 2015 [cited 2021 Jan 5]. Available from: <https://www.residencydatabase.com/belgium/>. |
| Croatia | 5. Public Health in Croatia EURONET MRPH [cited 2021 Jan 5]. Available from: <http://euronetmrph.org/residency-croatia/>.  6. MEDICALMOBILITY. Croatia: MEDICALMOBILITY.EU; [cited 2021 Jan 5]. Available from: <https://www.medicalmobility.eu/croatia>.  7. Specialist Programs: University of Zagreb, School of Medicine; [cited 2021 Jan 5]. Available from: <https://mef.unizg.hr/en/admission/postgraduate-programs-of-studies/specialist-studies>. |
| Czech Republic | personal communication, Oct 5, 2017  8. MEDICALMOBILITY. Czech Republic: MEDICALMOBILITY.EU; [cited 2021 Jan 5]. Available from: Medical Residency Database. |
| Germany | 9. Bogdanyova S, Lermann J, de Sousa Mendes M, Schott S. Becoming a resident in Germany: an experience-based practical guideline. Arch Gynecol Obstet. 2015;291(2):457-60.  10. Drossard S. Structured surgical residency training in Germany: an overview of existing training programs in 10 surgical subspecialties. Innov Surg Sci. 2019;4(1):15-24. |
| Greece | 11. MEDICALMOBILITY. Greece: MEDICALMOBILITY.EU; [cited 2021 Jan 5]. Available from: <https://www.medicalmobility.eu/greece>.  12. Zografos GN. TRAINING IN SURGERY IN GREECE [cited 2021 Jan 5]. |
| Hungary | 13. Surgical residency program in Hungary: SurgTTT; [cited 2021 Jan 5]. Available from: <http://cirugiaminimamenteinvasiva.es/SurgTTT-eLearning/index.php/en/mod4/surgical-residency-program-in-hungary?limitstart=0>.  14. Resident Training: Semmelweis University; [cited 2021 Jan 5]. Available from: <https://semmelweis.hu/english/education/resident-training/>. |
| Latvia | 15. MEDICALMOBILITY. Latvia: MEDICALMOBILITY.EU; 2014 [cited 2021 Jan 5]. Available from: <https://www.medicalmobility.eu/latvia>. |
| Lithuania | 16. MEDICALMOBILITY. Lithuania: MEDICALMOBILITY.EU; [cited 2021 Jan 5]. Available from: <https://www.medicalmobility.eu/lithuania>.  17. Residency: Vilnius University; [cited 2021 Jan 6]. Available from: <https://www.vu.lt/en/studies/residency>. |
| Luxembourg | Personal communication, Oct 20, 2017 |
| Sweden | 18. Specialistutbildningsfrågor SAfP-o. Läkares vidareutbildning i de nordiska länderna. 2010 |
| Denmark | 19. Schelde Holde J. Ansættelsesprocedure og forretningsorden. Danske Regioner; 2017.  20. Vejledning om ansøgning til hoveduddannelsesforløb i speciallægeuddannelsen i kirurgi 2021-1. Sekretariatet for lægelig videreuddannelse ØST.  21. Ansættelsessamtaler: Sekretariatet for lægelig videreuddannelse ØST; [updated May 15 2019; cited 2021 Jan 6]. Available from: <https://www.laegeuddannelsen.dk/speciallaegeuddannelsen/ansaettelse-i-uddannelsesforloeb/ansaettelsessamtaler.aspx>. |
| Netherlands | Personal communication, Oct 3, 2017 |
| Bulgaria | 22. Postgraduate Medical Study in Bulgaria: Study Medicine Europe; [cited 2021 Jan 5]. Available from: <https://www.studymedicineeurope.com/medical-specialties-bulgaria>. |
| Cyprus | 23. Medical Doctor´s Courses for Specialisation: Ministry of Health; [updated 04/01/2021. Available from: <https://www.moh.gov.cy/moh/moh.nsf/page54_en/page54_en?OpenDocument>. |
| Estonia | 24. Faculty of medicine UoT. Postgraduate specialist medical training University of Tartu [cited 2021 Jan 5]. Available from: <https://meditsiiniteadused.ut.ee/en/postgraduate-specialist-medical-training> |
| Finland | 25. MEDICALMOBILITY. Finland: MEDICALMOBILITY.EU; [Available from: <https://www.medicalmobility.eu/finland>.  26. Lastenkirurgia, erikoislääkäri: studieinfo.fi; [cited 2021 Jan 6]. Available from: <https://studyinfo.fi/app/#!/korkeakoulu/1.2.246.562.17.75997773173>. |
| France | 27. Tiret E. Surgical training in France. The Bulletin of the Royal College of Surgeons of England. 2008;90(2):54-5.  28. MEDICALMOBILITY. France: MEDICALMOBILITY.EU; [cited 2021 Jan 6]. Available from: <https://www.medicalmobility.eu/france>. |
| Ireland | 29. Royal College of Surgeons in Ireland SA. National Surgical Training Programme – Checklist 2018 Intake. Royal College of Surgeons in Ireland, RCSI; 2017.  30. Traynor O. ST1 Interviews 2018. Royal College of Surgeons in Ireland, RCSI; 2017. |
| Italy | 31. Ferrara PDP, Viola. Residency access in Italy: how did it change? : EURONET MRPH; 2018 [cited 2021 Jan 5]. Available from: Residency access in Italy: how did it change? . |
| Malta | Personal communication, Oct 22, 2017  32. MEDICALMOBILITY. Malta: MEDICALMOBILITY.EU; [cited 2021 Jan 6]. Available from: <https://www.medicalmobility.eu/malta>. |
| Poland | Personal communication, Oct 24, 2017  33. Wojciech Stefan Z, Zbigniew W, Mariusz R, Andrzej Ż, Mateusz J, Bartosz K, et al. Specialty training system in Poland in 2011-2018 according to the Centre of Postgraduate Medical Education register data. Wiedza Medyczna. 2020;2(1). |
| Portugal | 34. MEDICALMOBILITY. Portugal: MEDICALMOBILITY.EU; [cited 2021 Jan 6]. Available from: <https://www.medicalmobility.eu/portugal>.  35. Ramalho M. Medical Residence in Portugal: complete guide for foreign doctors: Atlantic Bridge; [cited 2021 Jan 6]. Available from: <https://atlanticbridge.com.br/en/residencia-medica-em-portugal-guia-completo-para-medicos-estrangeiros/>.  36. Doctors: Serviço Nacional de Saúde; [cited 2021 Jan 6]. Available from: <http://www.acss.min-saude.pt/category/professional/doctors/#tab_medical-internship>. |
| Romania | 37. Postgraduate Medical Study In Romania: Study in Romania; [cited 2021 Jan 5]. Available from: <https://www.studyinginromania.com/medical-studies-in-romania.html>. |
| Slovenia | 38. Šapec T. Postgraduate medical training in Slovenia 2016 [cited 2021 Jan 6]. Available from: <http://www.enmca.eu/system/files/postgraduate_medical_training_in_slovenia_2016_enmca_berlin.pdf>. |
| Spain | Personal communication, Oct 19, 2017  39. Freire JM, Infante A, de Aguiar AC, Carbajo P. An analysis of the medical specialty training system in Spain. Hum Resour Health. 2015;13:42. |
| UK | 40. CORE SURGICAL TRAINING - CT1. Health Education England.  41. Applicant guidance: Health Education England; [cited 2021 Jan 6]. Available from: <https://specialtytraining.hee.nhs.uk/Recruitment/Application-guidance>.  42. Surgical selection in the UK: Joint Committee on Surgical Training; [cited 2021 Jan 6]. Available from: <https://www.jcst.org/introduction-to-training/selection-and-recruitment/>.  43. Joint Commitee on Surgical training. Overview of Selection Process [PowerPoint]2019. |
